# Impact of 2013 south Asian haze crisis: study of physical and psychological symptoms and perceived dangerousness of pollution level

**DOI:** 10.1186/1471-244X-14-81

**Published:** 2014-03-19

**Authors:** Roger C Ho, Melvyn W Zhang, Cyrus S Ho, Fang Pan, Yanxia Lu, Vijay K Sharma

**Affiliations:** 1 Southeast Asian Haze Research Consortium, Jinan, China; 2 Institute of Medical Psychology, School of Medicine, Shandong University, Jinan, China

## Abstract

**Background:**

The widespread forest fires in Indonesia in June 2013 led to widespread haze to neighbouring countries. This is the first study in the medical literature reporting the acute physical and psychological symptoms of the general population during a haze crisis. We evaluated the factors that are associated with psychological stress of haze exposure.

**Methods:**

This study was conducted between June 21 to June 26, 2013. Participants were recruited by an online recruitment post and snowball sampling techniques. Participants were required to complete an online survey which was composed of demographics questionnaire, physical symptom checklist, perceived dangerous Pollutant Standard Index (PSI) value and views on the N-95 mask and the Impact of Event Scale-Revised (IES-R).

**Results:**

A total of 298 participants returned the completed study questionnaire. The respondents reported a mean number of 4.03 physical symptoms (S.D. = 2.6). The five most common physical symptoms include mouth or throat discomfort (68.8%), nose discomfort (64.1%), eye discomfort (60.7%), headache (50.3%) and breathing difficulty (40.3%). The total IES-R score was 18.47 (S.D. = 11.69) which indicated that the study population experienced mild psychological stress but not to the extent of acute stress reaction syndrome. The perceived dangerous PSI level and number of physical symptoms were significantly associated with the mean intrusion score, mean hyper-arousal score, total mean IES-R score and total IES-R score (p < 0.05).

**Conclusions:**

Our findings suggest that a haze crisis is associated with acute physical symptoms and mild psychological stress. The number of physical symptoms and the perceived dangerous PSI values are important factors associated with psychological stress.

## Background

The seasonal haze that afflicts large parts of Southeast Asia especially in the dry periods has drawn much international attention in view of the extensive health, socioeconomic and political impacts on the Association of Southeast Asian Nations (ASEAN) countries (ASEAN secretariat [1]). The countries usually affected include Singapore, Malaysia, Brunei, Southern Thailand and Indonesia. Widespread forest fires have been a regular event in Sumatra and Kalimantan since the early 1900s with the first serious episode occurring in 1997 when farmers adopted the ‘slash- and-burn’ technique of clearing land (1802 square kilometres (km^2^) to 2840 km^2^) for agricultural usage [2]. The burning of carbon-rich peatland would send off acrid smoke, dust and dry particles (2.5 micrometers or smaller) into the atmosphere thereby forming haze. Furthermore, recurrence of the El Nino-Southern Oscillation (ENSO) phenomenon has also contributed to the particularly dry season and intensified the pervasiveness of forest fires. Negative impacts associated with haze include increased emergency room attendance for respiratory tract symptoms [3,4], reduction in human productivity as a result of sick leave due to doubling of the number of asthma cases, inefficiency in manufacturing and outdoor construction, increasingly hazardous aviation condition and reduction in tourism [5]. As a result, the estimated economic loss attributed to the effects of the 1997 and 2006 haze crisis was estimated to US$9 million and US $50 million respectively [6,7].

**Figure 1:**
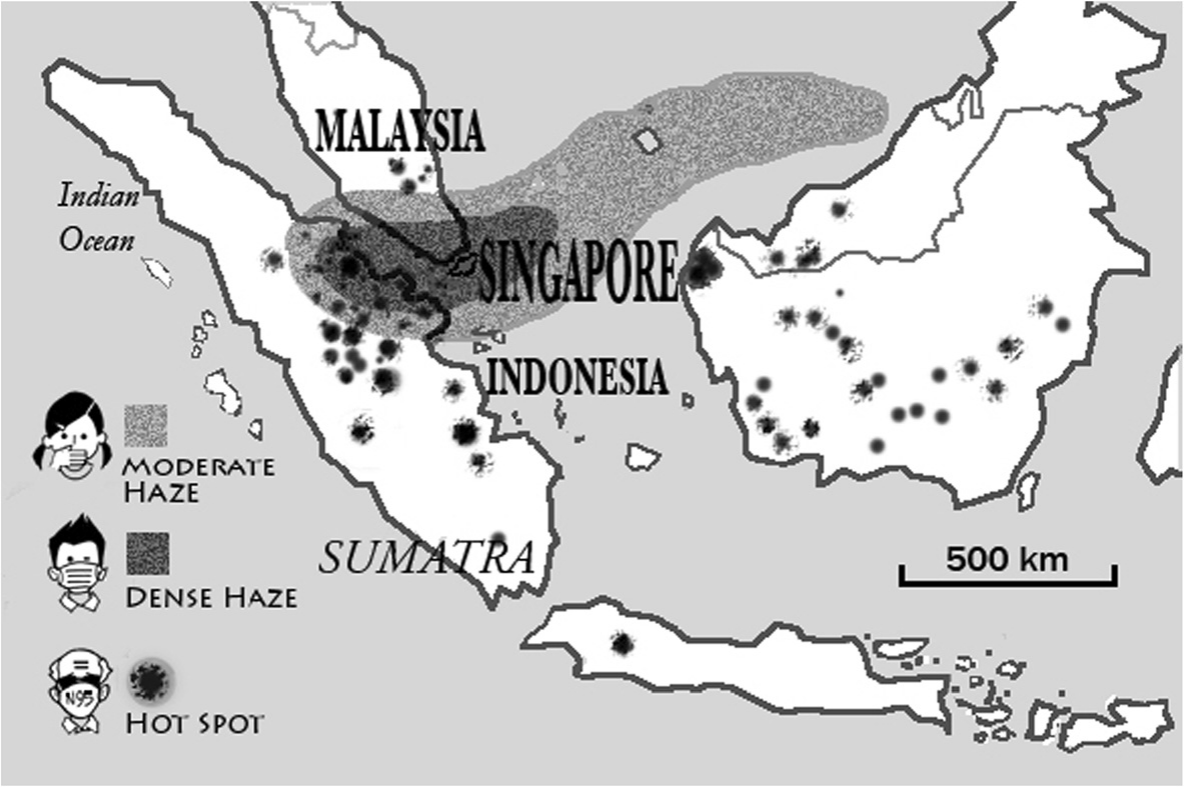
A geographical schematic map illustrating the density of haze over Southeast Asia during the 2013 haze crisis.

Since then, Southeast Asia has been experiencing the negative impacts of this recurrent problem, especially in early months of 1998, mid 1999 and 2006. In June 2013, Southeast Asia experienced its worst haze crisis. The chronological development of haze is summarized as follows. In early June, 2013, the haze was created by palm oil plantations and farmers in Indonesia who adopted the ‘slash- and-burn’ technique to prepare land for agricultural use. The National Environmental Agency [8] of Singapore used the Pollutant Standard Index (PSI) to describe air quality and the average PSI was calculated based on five locations of the Singapore Island (North, South, East, West and Central) which covered a land area of 714 km^2^. The PSI was developed by United States Environmental Protection Agency [9] and is based on the measurement of five pollutants including carbon monoxide, sulphur dioxide, nitrogen dioxide, ozone and fine particle < 10 micrometers (PM_10_) [10]. The PSI is a scale from 0 – 500 and the range is defined as follows: 0–50: good air quality; 51–100: moderate air pollution; 101–200: unhealthy; 201–300: very unhealthy; > 300: hazardous for health. On June 19, the haze spread eastwards and the air quality index started to deteriorate gradually. The PSI reached a hazardous level of 321 at 10 pm on June 19 2013 and the highest level of 401 on June 21. This led to a massive rush amongst Southeast Asians to stock up on the N-95 masks. Southeast Asians experienced the 1997 and 2006 haze crises and outbreak of the Severe Acute Respiratory Syndrome (SARS) in 2003 and they understand the use of N-95 masks when there is a serious health threat. During the 1997 haze crisis, the Ministry of Environment of Singapore recommended the general public to use the N-95 mask but not surgical mask because the N-95 mask is able to filter 95% of particles between 0.2 and 0.4 μm during period of intense air pollution [10]. Figure 1 shows a geographical schematic map illustrating the density of haze over Southeast Asia during the 2013 Haze crisis. Subsequently, the Indonesian government deployed military aircrafts for water bombings to put out fire in forest and plantations. The estimated burned area was around 2,000 hectares (20 km^2^) [11]. Since June 24, the PSI had fallen to moderate range but the government reminded its citizens to remain vigilant.

Schiffman [12] stated that health is a state of physical, mental and social well-being based on the World Health Organization’s definition. Gadit [13] defined ecology as an interaction between the living organisms and physical environment. Combining the two concepts together, degradation of an ecosystem may endanger physical and mental health of humans. The haze crisis was a temporary perturbation in the agricultural ecosystems and surrounding areas [12]. To our knowledge, there have not been previous studies that examined the immediate physical and psychological impact of haze on individuals. This study explored the immediate physical and psychological symptoms among the general population who experienced haze during the 2013 Southeast Asian Haze Crisis. This study also examined factors associated with the severity of acute physical and psychological symptoms.

## Methods

This study used a cross-sectional design and the research participants in Singapore were recruited by snowball sampling through sponsored online recruitment posted on a commonly used online social network with a direct link to the online questionnaire and with the following key words (haze, psychological impact, mental well-being, physical symptoms) during the week, June 21 to June 26, 2013 after the PSI level started to rise on June 21 and it was not certain how long the haze would last. The inclusion criteria for this study included participants who were (1) above the age of 17 years, (2) able to read English, (3) currently living in Singapore during the time of the study, (4) exposed to the environmental air and (5) able to demonstrate computer usage literacy. The Southeast Asian Haze Research Consortium worked with experts in Shandong University to set up the online survey. The Institutional Review Board of the Department of Medicine, Shandong University, People’s Republic of China approved this study, and the participants consented online before participation.

The online questionnaire was written in English and comprised of four sections, which acquired information relating to (1) demographics, (2) physical symptoms experienced during haze, (3) personal views on the of dangerous PSI value and usefulness of the N-95 masks and (4) the Impact of Event Scale - Revised (IES-R). The demographics questionnaire was comprised of 7 items in total, and was used to acquire the baseline characteristics of the participants partaking in the study, such as gender, age, ethnicity, marital status, level of education, occupation and most importantly, presence or absence of chronic medical illnesses. The questionnaire on physical symptoms inquired the presence or absence of the following physical symptoms: mental slowing, headache, dizziness, eye discomfort, nose discomfort, mouth and throat discomfort, breathing difficulty, heart pain or chest pain, nausea and vomiting, gastric or abdominal discomfort, slowness in movement and muscle ache or pain. The participants rated on the range of self-perceived dangerous PSI values *(Can you state the level of PSI (0–500) which you find dangerous to your health?)*, personal possession and perceived usefulness of the N-95 mask on 5-point Likert scales. The Impact of Event Scale- Revised (IES-R) is a 22-item self-administered questionnaire that has been well validated for determining extent of stress reaction after exposure to stressful circumstances, within one week of exposure across different cultural groups [14,15]. Each item on the scale was administered via a 5-point frequency scale (0 - Not at all, 1 - A little bit, 2 - Moderately, 3 - Quite a bit, 4 - Extremely) and higher scores indicate higher level of psychological stress. The IES-R provides three sub-scores, the mean avoidance, intrusion and hyperarousal scores, a total mean IES-R score (divided by the total number of items) and a total IES-R score (without division by the total number of items). A total IES-R score equal to or more than 33 signifies the likely presence of acute stress reaction syndrome [15,16].

All statistical analyses were performed using the SPSS statistical package program version 16.0 for Windows (SPSS Inc., Chicago, IL, USA). Categorical variables were expressed by number (N) and percentage (%). Continuous variables were expressed as mean ± standard deviation (SD). Chi-square analyses were used to compare demographic data, personal views on the N-95 mask and presence of physical symptoms across two groups based on gender and perceived dangerous PSI value. The participants were classified into two groups based on low or high perceived dangerous PSI value. The average (250) of the minimum (0) and maximum (500) PSI value was used as a cut-off (i.e. < 250 vs > 250). Using subscale of IES-R and total IES-R score as dependent variables, univariate linear regression were performed to identify factors associated with psychological stress during the haze crisis. Statistical significance was set at *p* < 0.05 for all analyses.

## Results

A total of 298 respondents participated in this study and the overall response rate was 87%. Table 1 summarizes the demographic characteristics of the 298 respondents. Majority of respondents (70.5%) were between the age of 18 to 29 years; 23.8% were married and only 7.3% had chronic medical illness. Majority of respondents (87.2%) were Chinese; 50.3% were students; 22.2% worked in an indoor setting and 27.5% worked in an outdoor setting.

**Table 1:** Demographic characteristics, perception of dangerous PSI level and availability of N-95 masks, physical symptoms and psychological stress of the haze crisis (n = 298)

**Item**	**Total sample (N = 298)**	**Men (120)**	**Women (178)**	**p-value**	**Perceived dangerous PSI value < 250 (178)**	**Perceived dangerous PSI value > =250 (120)**	**p-value**
	**N%**	**N%**	**N%**		**N%**	**N%**	
Age							
• 18–29 (210)	210(70.5)	82(71.9)	128(68.3)	0.507	126(71.2)	84(70.0)	0.826
• 30–69 (88)	88(29.5)	38(28.1)	50(31.7)		51(28.8)	36(30.0)	
Ethnicity							
• Chinese	260(87.2)	99(82.5)	161(90.4)	0.044	164(92.1)	96(80.0)	0.002
• Indian, Malay, other ethnicities	38(12.8)	21(17.5)	17(9.6)		14(7.9)	24(20.0)	
Marital status							
• Married	71(23.8)	31(25.8)	40(22.5)	0.297	39(21.9)	32(26.7)	0.345
• Others: Single, divorced, widow	227(76.2)	89(74.2)	138(77.5)		139(78.1)	88(73.3)	
Occupation							
• Students	150(50.3)	65(54.2)	85(47.7)	0.486	88(49.4)	62(51.7)	0.893
• Working indoor	66(22.2)	23(19.2)	43(24.2)		41(23.1)	25(20.8)	
• Working outdoor	82(27.5)	32(26.6)	50(28.1)		49(27.5)	33(27.5)	
Presence of chronic medical illness	20(7.3)	8(7.2)	12(7.4)	0.950	13(8.0)	7(6.4)	0.616
Personal possession of N—95 mask						\	
• Inadequate –very inadequate	194(66.0)	72(61.0)	122(69.3)	0.141	117(67.2)	77(64.2)	0.584
• Just enough – very adequate	100(34.0)	46(39.0)	54(30.7)		57(32.8)	43(35.8)	
Perceived usefulness of N-95 mask							
• Useless – absolutely useless	15(5.1)	11(9.4)	4(2.3)	0.007	9(5.1)	6(5.1)	0.996
• Of some use – extremely useful	278(94.9)	106(90.6)	172(97.7)		167(94.9)	111(94.9)	
**Physical symptoms**							
Mental slowing	82(27.5)	40(33.3)	42(23.6)	0.065	53(29.8)	29(24.2)	0.288
Headache	150(50.3)	90(50.6)	60(50.0)	0.924	97(54.5)	53(44.2)	0.080
Dizziness	74(24.8)	25(20.8)	49(27.5)	0.190	51(28.7)	23(19.2)	0.063
Eye discomfort	181(60.7)	63(52.5)	118(66.3)	0.017	111(62.4)	70(58.3)	0.485
Nose discomfort	191(64.1)	84(70.0)	107(60.1)	0.081	122(68.5)	69(57.5)	0.051
Mouth or throat discomfort	205(68.8)	78(65.0)	127(71.3)	0.246	135(75.8)	70(58.3)	0.001
Breathing difficulty	120(40.3)	42(35.0)	78(43.8)	0.128	71(39.9)	49(40.8)	0.870
Heart pain or chest pain	32(10.7)	16(9.0)	16(9.0)	0.235	24(13.5)	8(6.7)	0.062
Nausea or vomiting	30(10.1)	17(9.6)	13(10.8)	0.718	24(13.5)	6(5.0)	0.017
Gastric or abdominal discomfort	48(16.1)	16(13.3)	32(18.0)	0.285	32(18.0)	16(13.3)	0.285
Slowness in movement	51(17.1)	20(16.7)	31(17.4)	0.866	34(19.1)	17(14.2)	0.267
Muscle ache or pain	38(12.8)	15(12.5)	23(12.9)	0.915	19(10.7)	19(15.8)	0.190
Total number of physical symptoms	4.03(2.60)	3.93(2.74)	4.10(2.51)	0.586	4.34(2.61)	3.58(2.53)	0.012
**Psychological stress**							
Mean avoidance score mean ± (SD)	0.71(0.50)	0.70(0.52)	0.71(0.49)	0.779	0.71(0.47)	0.70(0.55)	0.779
Mean intrusion score mean ± (SD)	0.96(0.63)	0.94(0.62)	0.98(0.64)	0.671	1.03(0.63)	0.87(0.61)	0.029
Mean hyper-arousal score mean ± (SD)	0.85(0.74)	0.84(0.74)	0.86(0.75)	0.889	0.94(0.73)	0.72(0.76)	0.013
Total mean IES-R score mean ± (SD)	0.84(0.53)	0.84(0.74)	0.85(0.53)	0.740	0.89(0.51)	0.77(0.56)	0.047
Total IES-R score mean + (SD)	18.47(11.69)	18.19(11.73)	18.65(11.69)	0.740	19.57(11.18)	16.83(12.27)	0.047

PSI = Pollutant Standard Index.

The respondents reported a mean number of 4.03 physical symptoms (S.D. = 2.6) and the total IES-R score was 18.47 (S.D. = 11.69). The five most common physical symptoms include mouth or throat discomfort (68.8%), nose discomfort (64.1%), eye discomfort (60.7%), headache (50.3%) and breathing difficulty (40.3%). For the psychological stress, the mean intrusion score (mean = 0.96, SD = 0.63) was the highest, followed by mean hyper-arousal score (mean = 0.85, SD = 0.74) and the mean avoidance score (mean = 0.71, SD = 0.5) was the lowest. In the context of haze crisis, the intrusion scores represent recurrent thinking about haze, negative feelings associated with reminder of haze, dreams about haze and recurrent mental pictures of haze. The arousal scores represent irritability, easily startled responses, insomnia, poor concentration, on guard of haze and physical reactions after reminders of haze. The avoidance scores represent avoidance of feelings, reminders, recollections and discussion about the haze. When comparing the responses between male and female participants, women were more likely to report usefulness of the N-95 mask (X^2^ = 7.353, df = 1, p = 0.007) and the presence of eye discomfort (X^2^ = 5.718, df = 1, p = 0.017). There were no significant differences between men and women in demographics variables and scores on psychological stress. Respondents were further classified into two groups based on the perceived dangerous PSI value. Respondents who perceived lower PSI value (<250) as dangerous were more likely to come from other ethnic groups (X^2^ = 9.487, df = 1, p = 0.002); report the presence of mouth or throat discomfort (X^2^ = 10.236, df = 1, p = 0.001), nausea or vomiting (X^2^ = 5.697, df = 1, p = 0.017), higher number of physical symptoms (t = 2.522, df = 296, p = 0.012), higher mean intrusion score (t = 2.198, df = 296, p =0.029), higher mean hyper-arousal score (t = 2.488, df = 296, p = 0.013), higher total mean IES-R score (t = 1.990, df = 296, p = 0.047) and higher total IES-R score (t = 1.990, df = 296, p = 0.047).

Table 2 summarizes the factors associated with psychological stress during the haze crisis. The perceived dangerous PSI level was negatively associated with the mean intrusion score (B = -0.162, SE = 0.074, R^2^ = 0.016, p = 0.029), mean hyper-arousal score (B = -0.217, SE = 0.087, R^2^ = 0.020, p = 0.013), total mean IES-R score (B = -0.124, SE = 0.062, R^2^ = 0.013, p = 0.047) and total IES-R score (B = -2.734, SE = 1.374, R^2^ = 0.013, p = 0.047). The total number of physical symptoms was positively associated with the mean avoidance score (B = 0.048, SE = 0.011, R^2^ = 0.061, p < 0.001), mean intrusion score (B = 0.075,SE = 0.013, R^2^ = 0.095, p <0.001), mean hyper-arousal score (B = 0.130, SE = 0.015, R^2^ = 0.207, p <0.001), total mean IES-R score (B = 0.080, SE = 0.011, R^2^ = 0.153, p <0.001) and total IES-R score (B = 1.759, SE = 0.240, R^2^ = 0.153, p <0.001). The demographic variables, actual PSI score during the survey and views on the N-95 masks were not associated with psychological stress during the haze crisis (p > 0.05).

**Table 2:** Univariate regression analysis of factors which determined the psychological stress (n = 298)

	**Mean avoidance score**				**Mean intrusion score**				**Mean hyper-arousal score**				**Total mean IES-R score**				**Total IES-R score**			
	**B**	**SE**	**R**^**2**^	**p-value**	**B**	**SE**	**R**^**2**^	**p-value**	**B**	**SE**	**R**^**2**^	**p-value**	**B**	**SE**	**R**^**2**^	**p-value**	**B**	**SE**	**R**^**2**^	**p-value**
**Age**	0.028	0.064	0.001	0.663	-0.043	0.080	0.001	0.595	0.035	0.095	0	0.714	0.004	0.068	0	0.952	0.089	1.492	0	0.952
**Gender**	-0.017	0.059	0	0.779	-0.032	0.075	0.001	0.671	-0.012	0.088	0	0.889	-0.021	0.063	0	0.740	-0.460	1.383	0	0.740
**Ethnicity**	0.046	0.087	0.001	0.600	0.009	0.110	0	0.936	-0.031	0.130	0	0.810	0.011	0.092	0	0.902	0.250	2.033	0	0.902
**Marital status**	-0.004	0.068	0	0.958	0.092	0.086	0.004	0.284	0.064	0.101	0.001	0.526	0.050	0.072	0.002	0.493	1.093	1.591	0.002	0.493
**Occupation**	0.006	0.034	0	0.859	-0.009	0.043	0	0.837	0.036	0.051	0.002	0.480	0.009	0.036	0	0.809	0.193	0.796	0	0.809
**Presence of chronic medical illness**	0.097	0.118	0.003	0.410	-0.070	0.144	0.001	0.626	0.132	0.167	0.002	0.430	0.046	0.122	0.001	0.708	1.010	2.691	0.001	0.708
**Perceived dangerous PSI value**	-0.017	0.059	0	0.779	-0.162	0.074	0.016	0.029	-0.217	0.087	0.020	0.013	-0.124	0.062	0.013	0.047	-2.734	1.374	0.013	0.047
**The actual PSI value at the time of survey**	-0.001	0	0.012	0.055	0	0	0	0.907	0	0.001	0.003	0.358	0	0	0.004	0.290	-0.008	0.008	0.004	0.290
**Personal possession of N-95 mask**	0.030	0.061	0.001	0.621	-0.142	0.077	0.011	0.068	-0.160	0.092	0.010	0.082	-0.084	0.065	0.006	0.199	-1.849	1.435	0.006	0.199
**Perceived usefulness of N-95 mask**	0.045	0.133	0	0.733	0.173	0.167	0.004	0.300	0.230	0.198	0.005	0.247	0.142	0.141	0.003	0.314	3.130	3.102	0.003	0.314
**Total number of physical symptoms**	0.048	0.011	0.061	<0.001	0.075	0.013	0.095	<0.001	0.130	0.015	0.207	<0.001	0.080	0.011	0.153	<0.001	1.759	0.240	0.153	<0.001

IES-R = Impact of Event Scale - Revised.

## Discussion

Ostermann and Brauer [10] stated that there were very few studies which reported the impacts on physical health during the haze episodes. Besides physical symptoms, we assessed the psychological stress of a haze crisis, a temporary perturbation on the ecosystem and a period of uncertainty with hour-to-hour fluctuations of the PSI values. We were able to conduct this study through online survey to overcome the barrier of recruitment when the air quality was poor and people tended to stay at home. Our findings show that the perceived dangerous PSI value, not the actual PSI value and number of physical symptoms were associated with negative psychological impact during the haze crisis. Furthermore, the higher number of physical symptoms was associated with greater psychological stress. This finding suggests that reduction in the number of physical symptoms may reduce psychological stress during a haze crisis.

Haze contains increased concentrations of carbon-dioxide, carbon mono-oxide, other gases and the particulate material. Long et al. [17] studied the health impacts of burning agricultural residue on the population of Winnipeg, Canada and found that 42% of the population reported worsening respiratory symptoms as a result of the air pollution episode. The respiratory symptoms included cough, wheezing, chest tightness and shortness of breath. 20% of the population reported breathing difficulty. This study also found that female gender and smoking habits were important factors influencing susceptibility to respiratory symptoms during the Winnipeg air pollution crisis. Known hemodynamic influences of various constituents of the haze could have been responsible for other symptoms like headache, dizziness, chest pain, mental and physical slowing [18,19].

Some of the findings related to the psychological stress of haze warrant further thought and interpretation. The total IES-R score was 18.47 (SD = 11.69). A total IES-R score of 33 or over signifies the likely presence of acute stress reaction syndrome [15,16]. Our results suggest that the haze is not associated with acute stress reaction syndrome in the general population but it is associated with mild to moderate psychological stress. Intrusive symptoms such as recurrent thoughts, mental images and feelings about haze were more common than hyper-arousal and avoidance symptoms in the study population. The local government played a key role in reducing the psychological stress. On June 21, the local government took immediate measures to protect its citizens including hourly update of PSI value on television, cancellation of all school activities, offering medical subsidies to treat physical discomfort associated with haze and to provide 1 million free N-95 masks to the needy households [20]. Due to the adequate supply of N-95 masks, the personal possession and perceived usefulness of N-95 masks were not associated with psychological stress.

As there was limited number of studies on psychological stress during a haze crisis in Southeast Asia, the interpretation of our findings mainly rely on previous studies on infectious disease outbreak such as SARS in neighbouring countries. Our findings were similar to a study reported by Lee et al. [21] on the psychological responses of pregnant women during the SARS outbreak in Hong Kong in 2003. Lee et al. [21] reported that anticipatory worries were common among the pregnant women during the SARS outbreak; overestimation of risk led to higher level of anxiety and the levels of depression were similar between the SARS and pre-SARS outbreak cohorts. In this study, the inverse association between the perceived dangerous PSI values and mean scores of intrusion, hyper-arousal and overall psychological stress suggest that people who have lower threshold for health hazard are vulnerable to greater psychological stress when air quality deteriorates. Perceived dangerous PSI value used in this study or perceived air quality in general is an important indicator for air pollution research [22] because environmental acceptability by an individual can be different from objective measurements of pollutant levels [23]. For indoor pollutant research, Satish et al. [24] emphasized the need to measure perceived air quality as it is known to be associated with higher indoor pollutant levels, which in turn, associated with impaired work performance and increased health symptoms. A comparable study by Wargocki et al. [25] among office workers showed an increase in perceived freshness of air and general well-being, as well as a reduction in sensation of dryness of mouth and throat and difficulty in clear thinking after an increase of ventilation in simulated offices. For outdoor pollutant research, Valentić et al. [26] in a study of a population close to a big industrial pollutant producers reported that mental cognition with a tendency to overestimate the level and danger of pollution was associated with poor perception of physical health, despite the fact that the concentration of the pollutants were within legal ranges.

The low threshold for health hazard and the number of physical symptoms independently determine psychological stress and these factors may contribute to the anticipatory anxiety. Our study did not find a difference in the levels of psychological stress between respondents who filled the questionnaire during the periods with low and high PSI values. Lee et al. [21] found that people tended to receive more social support during the SARS outbreak. We did not measure social support in this study but it may be possible that people would receive more social support from their families during the haze outbreak as they tend to stay at home or participate in indoor activities with families.

There are several limitations in this study. First, the 2013 Southeast Asian haze crisis was a temporary perturbation on the ecosystem which lasted for one week. Therefore, the period of exposure to the haze was short. We could only study the acute physical symptoms and psychological stress. Our results cannot be generalized to sub-acute and long-term physical and psychological complications if the haze continues. This is also a common limitation in studies conducted during an outbreak of infection [21]. Second, this study has a cohort bias because the study population was mainly consisted of younger and educated individuals, recruited by snowball sampling which is a non-random sampling method. The participants were less likely to attend emergency department as a result of physical complications. Our participants were more informed about the ongoing haze, associated possible physical dangers as well as the steps taken by the government. These factors might have influenced their responses. Third, the list of physical symptoms was derived from general medical knowledge and it was not designed to detect specific physical symptoms associated with haze. Our results cannot be generalized to the ‘less-informed’ older population, those with lower education level and Asian population who do not understand English. Similarly, we could not study the health impacts on infants and children, especially those who reside in rural areas. Rukumnuaykit [27] found that rural infants in Indonesia born during the 1997 haze crisis experienced approximately 4.4 per cent increase in their infant mortality risks. Fifth, this was a cross-sectional study and described the physical symptoms and psychological stress of a population experiencing a haze crisis, but not able to determine cause-and-effect relationships between different variables. Nevertheless, conducting research, recruiting subjects and gathering data during the short period of a haze crisis was challenging, especially the study period included a weekend and people were less likely to be reachable by conventional method (e.g. face to face interview).

## Conclusions

This study highlights that even a short-term exposure to haze is associated with acute physical symptoms and mild psychological disturbances in healthy individuals. We hope that our results will raise the global awareness of the negative effects of haze on the physical and mental health of humans. Our findings provide guidance to the health authorities to focus on reducing physical symptoms, especially headache, eye, nose and throat discomfort and breathing difficulty, in order to reduce psychological stress during a haze crisis. It is important to note that the perceived dangerous PSI value, not the actual PSI level, is associated with negative psychological stress. Timely government actions, especially regular updating and forecasting of the haze situation, increasing the awareness and educating the general public can help in reducing psychological stress. Regional organization such as the ASEAN should formulate and reinforce policies to prevent future illegal burning in farms and plantations as a cheap way to prepare land for agricultural use.

## Competing interests

1. In the past five years have you received reimbursements, fees, funding, or salary from an organization that may in any way gain or lose financially from the publication of this manuscript, either now or in the future? Is such an organization financing this manuscript (including the article-processing charge)? NO.

2. Do you hold any stocks or shares in an organization that may in any way gain or lose financially from the publication of this manuscript, either now or in the future? NO.

3. Do you hold or are you currently applying for any patents relating to the content of the manuscript? Have you received reimbursements, fees, funding, or salary from an organization that holds or has applied for patents relating to the content of the manuscript? NO.

4. Do you have any other financial competing interests? If so, please specify. Non-financial competing interests NO.

5. Are there any non-financial competing interests (political, personal, religious, ideological, academic, intellectual, commercial or any other) to declare in relation to this manuscript? NO.

## Authors’ contributions

RCMH, VKS, MWBZ conceived of the study, and participated in its design and coordination of study and helped to draft the manuscript. MWZ & FP coordinated the online distribution of survey. FP prepared the protocol and sought ethics approval from Shandong University. CSH performed the literature review and wrote the introduction. YL and RCMH performed the statistical analysis and designed data presentation. All authors read and approved the final manuscript.

## Pre-publication history

The pre-publication history for this paper can be accessed here:

http://www.biomedcentral.com/1471-244X/14/81/prepub
